# Haplotype-resolved genome sequences of a springtail species, *Folsomia candida* (Collembola: Isotomidae)

**DOI:** 10.1038/s41597-026-07038-0

**Published:** 2026-03-28

**Authors:** Zhihong Zhan, Hao Yang, Jianfeng Jin, Feng Zhang

**Affiliations:** 1 Department of Entomology, College of Plant Protection, Nanjing Agricultural University, Nanjing, 210095 China; 2 State Key Laboratory of Agricultural and Forestry Biosecurity, College of Plant Protection, Nanjing Agricultural University, Nanjing, 210095 China; 3 College of Life Science, Xinyang Normal University, Xinyang, 464000 China

**Keywords:** Genome, Evolution

## Abstract

*Folsomia candida* (Collembola: Isotomidae) is one of the most important indicator species for soil animal studies and is widely distributed worldwide. Here, we utilized PacBio HiFi long reads, Oxford Nanopore Technologies (ONT) ultralong reads, Hi-C, Illumina short reads, and ONT long-read transcriptome sequencing to assemble and annotate the genome of *Folsomia candida*. Two gapless, telomere-to-telomere, haplotype-resolved genomes (HapA and HapB) were successfully assembled, with genome sizes of 228.62 and 228.45 Mb, respectively. The scaffold N50 sizes are 41.45 Mb for both haplotypes. We manually curated and annotated each gene on HapA, providing comprehensive annotation information for this haplotype. Gene annotation revealed a total of 26,329 protein-coding genes in HapA and 26,768 in HapB. These haplotype-resolved *Folsomia candida* genomes provide a high-quality genomic resource that enables future studies on soil arthropods and springtail genomics.

## Background & Summary

*Folsomia candida* (Collembola: Isotomidae) is a widely used model organism in soil ecotoxicology and environmental monitoring, owing to its global distribution, ease of laboratory cultivation, and established role in standardized soil toxicity assays^1,2^. As a representative springtail species, *F. candida* has long served as an indicator animal of soil quality and the effects of environmental stressors^3,4^. These features have made *F. candida* an important reference species in soil biology and environmental research.

The investigation of *F. candida* genomics began in 2017 with the first genome assembly, which comprised 221.7 Mb in 162 scaffolds, representing a significant milestone in springtail genomics^5^. Subsequently, several improved versions of assemblies have been published, enabling comparative analyses and providing useful foundations for ecological and evolutionary studies^2^. However, existing assemblies still contained gaps and did not revolve haplotypes, limiting further utilization for high-resolution genomic applications.

To address these limitations and provide a more complete *F. candida* genomic resource, we integrated PacBio HiFi, ONT ultralong reads, Hi-C data, Illumina short reads, and ONT long-read transcriptome data to generate new high-quality, haplotype-resolved reference genomes. By combining these datasets with our previous Hi-C data, we successfully assembled haplotype-resolved, telomere-to-telomere (T2T), and gapless genomes (HapA and HapB). The haplotype-phased genomes consist of 7 chromosomes for each haplotype, with genome sizes of 228.62 Mb (HapA) and 228.45 Mb (HapB), representing scaffold N50 values of 41.45 Mb for both haplotypes. We manually curated and corrected gene annotations on HapA, providing comprehensive annotation information. This T2T genome assembly delivers complete genomic information for *F. candida* and establishes a high-quality genomic framework for advancing soil arthropod research and comparative genomics studies.

## Methods

### Sample collection

A single *F. candida* female was taken from the laboratory of Nanjing Agricultural University, Dermark strain (FCDK), and then reared in a special container with peat soil for parthenogenic reproduction. Around 600 offspring, including the samples of PacBio HiFi, ONT ultralong, and ONT long-read transcriptome, were extracted from the single female reproductive population and washed via phosphate-buffered saline (PBS) solution for five minutes to eliminate any possible external pollutants. Specimens were then transferred into liquid nitrogen, frozen for at least 20 minutes, and kept at −80 °C for temporary storage until sequencing.

### Library construction and sequencing

Specimens’ genomic DNA (gDNA) was extracted using the FastPure® Blood/Cell/Tissue/Bacteria DNA Isolation Mini Kit (Vazyme Biotech Co., Ltd, Nanjing, China). High molecular weight (HMW) gDNA was sheared into 15 kb fragments using the MegaruptorTM device (Diagenode, Liege, Belgium) and purified using AMPurePB Beads. A PacBio HiFi 15 kb library was prepared using the SMRTbell Express Template Prep Kit 2.0, and the resulting library was sequenced on the PacBio Sequel II platform. For ultralong sequencing, HMW gDNA was prepared without shearing, and libraries were constructed using the Oxford Nanopore Technologies (ONT) Ligation Sequencing Kit, followed by sequencing on the PromethION platform. ONT long-read transcriptome libraries were constructed using the Direct RNA Sequencing Kit and sequenced on the PromethION platform. All library constructions and sequencing were performed by Berry Genomics (Beijing, China). Consequently, we newly obtained 161.40 Gb of sequencing data, including 28.00 Gb (123×) of PacBio HiFi reads, 14.30 Gb (63×) of ONT ultralong reads, and 24.90 Gb of ONT long-read transcriptome data (Table 1).

**Table 1:** Statistics of the sequencing data applied for *Folsomia candida*, T2T assembly, asterisks represent the new data obtained for this study.

Libraries	Insert sizes	Raw data (Gb)	Coverage (×)	Source
PacBio*	15Kb	28.00	122	NCBI(SRR35197880)
ONT ultralong*	100Kb	14.30	63	NCBI (SRR35197879)
Hi-C	350 bp	48.85	222	NCBI (SRR13435515)
RNA	350 bp	6.80	—	NCBI (SRR13452035)
RNA-ONT*	5Kb	25.40	—	NCBI (SRR35197878)

### Haplotype-resolved genome assembly

Raw PacBio HiFi reads were assembled into the primary assembly using Hifiasm v0.19.8^6^. The outcome HiFi data was then combined with ONT ultralong reads and Hi-C data using default parameters to separate each haplotype. Consequently, a total of 7 chromosomes were assembled for each haplotype, and N50 values were 41.45 Mb for each, respectively.

### Hi-C refinement and polishing

**Fig. 1:**
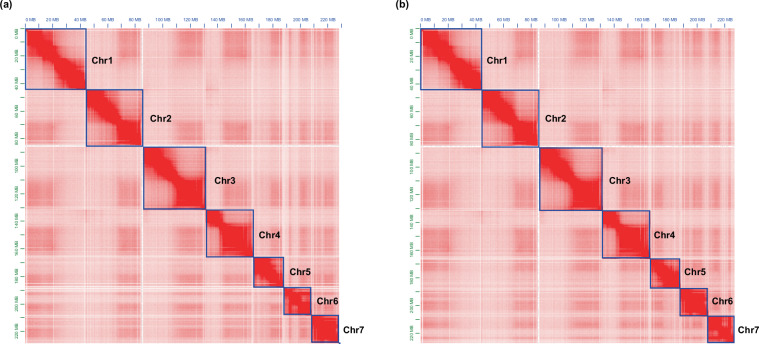
Genome-wide Hi-C contact heatmaps of the *Folsomia candida* assemblies. The heatmaps display normalized Hi-C interaction frequencies, with higher contact intensities shown as darker colors. Each heatmap illustrates the chromosomal organization and interaction patterns supporting the chromosome-scale assembly. Chromosomes are framed in blue, and contigs (if present) are framed in green. (**a**) Hi-C contact map for HapA, showing seven well-defined chromosome interaction domains with strong intra-chromosomal contacts and minimal inter-chromosomal signal. (**b**) Hi-C contact map for HapB, similarly exhibiting seven chromosome-scale interaction domains consistent with a complete haplotype assembly.

We applied the Hi-C data for genome assembly. Raw Hi-C data were processed for quality control to remove duplicates with Chromap v.0.2.5-r473^7^. The clean Hi-C data were then used to align the primary assembly for haplotype identification and division. By applying YaHS v1.2^8^ and Juicer v1.6.2^9^, contigs were anchored and oriented onto chromosomes successfully. We manually checked outcome results under Juicebox v.1.11.08^10^, and corrected any potential assembly errors to generate the final Hi-C assembly (Fig. 1).

To ensure high-quality genome assembly, we applied a comprehensive contamination detection and removal pipeline targeting sequences from humans, bacteria, viruses, and plants. Primary contamination screening was performed using MMseq. 2 v11.28^11^ with BLASTN-like searches against the UniVec database, while vector contamination was specifically investigated through BLASTN searches (BLAST + v2.11.029). Our stringent filtering criteria automatically removed sequences showing >90% similarity to known contaminants, while sequences with 80–90% similarity underwent additional verification through online BLASTN analysis against the NCBI nucleotide database. The final assembled genome was checked through NCBI’s automatic contamination screening pipeline, which revealed no significant contaminant sequences Table 2.

**Table 2:** Chromosome lengths of *Folsomia candida*’s two haplotypes and the previous assembly genome, FCDK.

HapA		HapB	FCDK	
Chromosome Number	Length (Mb)	Length (Mb)	Chromosome Number	Length (Mb)
1	44.61	44.63	3	43.32
2	41.45	41.45	4	38.47
3	45.87	45.70	1	44.87
4	34.62	34.61	7	32.24
5	21.62	21.62	5	20.37
6	20.34	20.34	6	18.19
7	20.08	20.08	2	19.19

The final genome assembly comprised two distinct gapless haplotypes, and we named them HapA (228.62 Mb) and HapB (228.45 Mb), respectively. Both haplotypes exhibited larger genome size and fewer contigs (Table 3), representing our high-quality T2T haplotype-resolved outcome.

**Table 3:** Genome assembly and annotation statistics for Haplotype-resolved genomes and the previous assembly genome, DK strain (FCDK).

Name	HapA	HapB	FCDK
Genome assembly			
Size (Mb)	228.62	228.45	219.07
Number of contigs	7	7	75
Number of chromosomes	7	7	7
Scaffold N50 length (Mb)	41.45	41.45	38.47
GC (%)	37.71	37.71	37.48
BUSCO completeness (%)	97.5	97.5	97.6
Protein-coding genes			
Number	26,329	26,768	25,139
Mean gene length (bp)	475.5		470.2
Repetitive elements			
Size (%)	24.15	24.20	22.6
DNA transposons (%)	4.43	5.39	4.23
SINEs (%)	0.02	0.01	0
LINEs (%)	0.9	1.09	1.46
LTRs (%)	2.05	2.28	2.17
Unclassified (%)	14.92	13.69	13.34
ncRNAs			
rRNA	135	142	70
miRNA	33	32	34
snRNA	54	52	54
ribozyme	4	4	6
tRNA	113	122	115
lncRNA	2	2	2
Others	111	111	108
Total number of ncRNAs	452	465	396

### Telomere and centromere detection

Telomeres for each haplotype were detected by identifying the animal telomeric repeat sequences (AATCCG/AAATTT/AAACCT). We performed the *F. candida* telomere identification by using the TeloExplorer model of quarTeT v1.2.5^12^. A total of 14 telomeres (7 chromosomes) for each haplotype were eventually recognized (Fig. 3). For centromere detection, we utilized RepeatObserverV1^13^ on both haplotypes to identify centromeric tandem repeat clusters. Consequently, 7 centromeres were identified for each haplotype (Fig. 3).

**Fig. 2:**
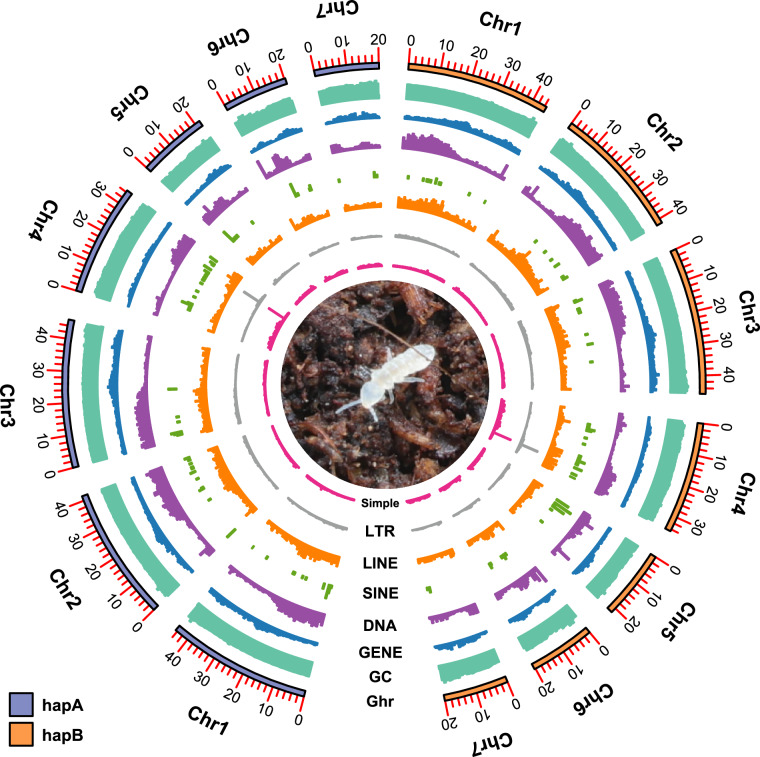
Genome characteristics of *Folsomia candida*. Circos plot showing the genomic characters of *F. candida* from outer to inner: chromosome length (Chr) (Mb), the density of GC content (GC), the density of protein-coding genes (GENE), the density of TEs (DNA, SINE, LINE, and LTR), and simple repeats (Simple). Blue chromosomes represent HapA, and orange chromosomes represent HapB (The sliding window size is counted for every 10 kb).

**Fig. 3:**
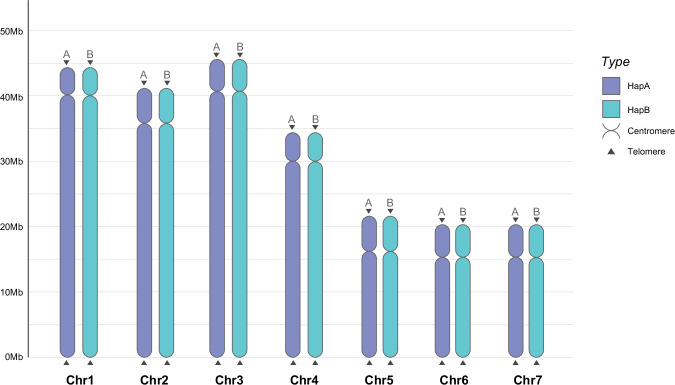
Telomere and centromere detection of two haplotype-resolved genomes. Darker color chromosomes represent HapA, and brighter color chromosomes represent HapB. All telomeres were marked with triangle symbols, and all centromere positions were exhibited by the bar graph.

### Collinearity analysis

**Fig. 4:**
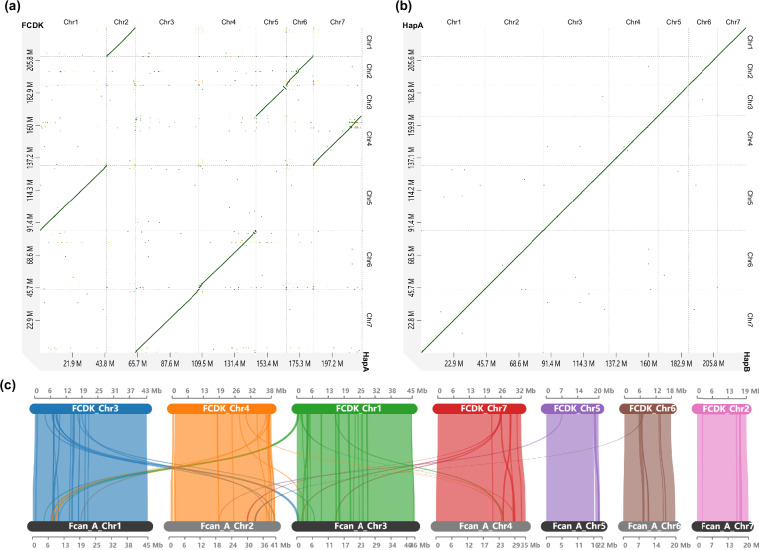
Genome collinearity analysis of *Folsomia candida*. (**a**) Synteny test between HapA (right) and the previous genome version, FCDK (upper); (**b**) Synteny test between HapA (upper) and HapB (right); (**c**) Collinearity analysis between HapA (below) and FCDK (upper), genes with darker colors representing the assembly errors of FCDK.

Genome collinearity analysis between our two haplotypes and the previous assembly genome FCDK was performed using a dual-validation approach. Initially, D-Genies^14^ was employed to provide a comprehensive whole-genome dotplot visualization, offering an intuitive overview of large-scale syntenic relationships and potential structural variations between assemblies (Fig. 4a,b). To further validate these findings and obtain more detailed syntenic block information, we performed complementary collinearity analysis between HapA and FCDK using MCScanX^15^, which utilizes gene-based collinearity detection algorithms. The results were subsequently visualized through SynVisio^16^ (Fig. 4c). Consequently, collinearity analysis between HapA and FCDK maintained the consistent result, and exhibited the previous assembly errors in FCDK (Fig. 4c). Both analysis approaches demonstrated consistent results, revealing remarkable collinearity between our haplotype-resolved assembly and the FCDK genome. Based on these unambiguous syntenic relationships, we further established a clear one-to-one correspondence between HapA chromosomes and the FCDK chromosome identifiers. This mapping is summarized in Table 4 and is provided to facilitate cross-reference between assemblies and ensure consistency with previous literature.

**Table 4:** One-to-one correspondence between chromosomes in the HapA assembly and those in the FCDK reference genome.

Hap A	FCDK
Chr 1	Chr 3
Chr 2	Chr 4
Chr 3	Chr 1
Chr 4	Chr 7
Chr 5	Chr 5
Chr 6	Chr 6
Chr 7	Chr 2

This mapping is provided to ensure consistency with previous literature and to facilitate cross-reference between assemblies.

### Gene prediction and annotation

Three strategies were combined for protein-coding genes (PCGs) annotation for *F. candida* HapA, including transcriptome-assisted prediction, homology-based proteins, and *ab initio* gene predictions. Transcribed RNA alignment prediction was performed by HISAT2 v2.2.1^17^, and relative production was then used as a genome-guided assembly by StringTie v2.1.6^18^. We used BRAKER v3.0.3^19^ to acquire the *ab initio* gene predictions with the combination of GeneMark-ETP^20^ and Augustus v3.4.0^21^, and automatically trained based on RNA sequence alignments and reference proteins obtained from the OrthoDB v11 database. The GeMoMa v1.9^22^ was applied to protein-homology alignments from seven arthropod species, including *Drosophila melanogaster* (GCA_000001215.4^23^), *Tribolium castaneum* (GCF_000002335.3^24^), *Apis mellifera* (GCA_003254395.2^25^), *Bombyx mori* (GCF_030269925.1^26^), *Daphnia magna* (GCF_003990815.1^27^), *Folsomia candida* (GCF_002217175.1^5^), and *Sinella curviseta* (GCA_004115045.4^28^). Results from BRAKER and GeMoMA were finally combined and applied as the *ab initio* input for MAKER v3.01.03^29^ to generate a GFF3 file.

To improve the accuracy of the annotation results, we manually curated each gene’s length and structure on all chromosomes using IGV-GSAman, integrating evidence from *ab initio* prediction, protein homology, and transcriptome data. The manual curation followed strict validation criteria: 1) A CDS was considered valid only when supported by at least two types of evidence among *ab initio* prediction, protein homology, and transcriptome data; 2) A CDS was required to have supporting evidence from either protein homology or transcriptome data (or both). Finally, 26,329 protein-coding genes were annotated on HapA, with the mean protein length of 475.5 aa. The mean gene length was 4,348.8 bp with an average of 6.8 exons per gene (Table 5). We applied the HapA annotation result as the reference, using LiftOn^30^ to automatically annotate the HapB and get 26,768 protein-coding genes.

**Table 5:** Summary statistics of genome annotations in the HapA.

Structure annotation	
Number of protein-coding genes	26,329
Number of predicted protein sequences	26,345
Mean protein length (aa)	475.5
Mean gene length (bp)	4,348.8
Gene ratio (%)	50.08
Number of exons per gene	6.8
Mean exon length (bp)	286.3
Exon ratio (%)	22.63
Number of CDSs per gene	6.5
Mean CDS length (bp)	219.0
CDS ratio (%)	16.47
Number of introns per gene	5.8
Mean intron length (bp)	406.9
Intron ratio (%)	27.45
Function annotation	
Number of genes matching Uniprot records	25,309
Number of genes labeled as “Uncharacterized protein”	5,114
Number of genes labeled as “unknown function”	1,040
Number of genes with InterProScan annotations	18,184
Number of genes with GO items from InterProScan annotations	10,533
Number of genes with KEGG pathway items from InterProScan annotations	0
Number of genes with eggNOG annotations	17,784
Number of genes with GO items from eggNOG annotations	12,165
Number of genes with Enzyme Codes (EC) from eggNOG annotations	4,719
Number of genes with KEGG ko terms from eggNOG annotations	11,365
Number of genes with KEGG pathway terms from eggNOG annotations	7,291
Number of genes with COG Functional Categories from eggNOG annotations	16,889
Number of genes with GO items (combining InterProScan and eggNOG results)	14,974

### Repetitive sequence annotation

We performed the repetitive sequence annotation, including tandem repeats and interspersed repeats (transposable elements, TEs) for both haplotype genomes. A *de novo* specific repeat library was constructed for HapA and HapB by RepeatModeler v2.0.4^31^. This library was further combined with the RepBase-20230909^32^, and repeat elements were recognized and masked by RepeatMasker v.4.1.5^33^ by aligning the custom library. For HapA, results demonstrated 55.22 Mb of repetitive sequence (24.15%), including DNA (4.43%), LINE (0.9%), LTR (2.05%), SINE (0.02%), and Unclassified elements (14.92%). For HapB, we found 55.29 Mb (24.20%) of repetitive sequence, comprising DNA (5.39%), LINE (1.09%), LTR (2.28%), SINE (0.01%), and Unclassified elements (13.69%). Both HapA and HapB exhibited similar repetitive element results, which reconfirmed our haplotype-resolved genomes’ accuracy (Fig. 2).

### Non-coding RNA annotation

Non-coding RNAs (ncRNAs) and transfer RNA (tRNA) of two haplotypes in *F. candida* were detected and identified by Infernal v1.1.4^34^ and tRNAscan-SE v2.0.9^35^. As a result, 452 ncRNAs were identified in HapA, including two long non-coding RNAs, 4 ribozymes, 33 microRNAs, 54 small nuclear RNAs, 111 other ncRNAs, 113 tRNAs, and 135 rRNAs. 465 ncRNAs were found in HapB, including two long non-coding RNAs, 4 ribozymes, 32 microRNAs, 52 small nuclear RNAs, 111 other ncRNAs, 122 tRNAs, and 142 rRNAs (Table 3).

### Functional gene annotation

The functional gene annotation was performed on HapA by searching against the UniProtKB database (SwissProt and TrEMBL, version 20221130) using Diamond v2.0.11.1^36^. Protein domain identification and functional annotation were conducted using eggNOG-mapper v2.1.9^37^ and InterProScan 5.60–92.0^38^ for Gene Ontology (GO) and KEGG pathway annotation analysis. Five databases, including Pfam^39^, SMART^40^, Superfamily^41^, Gene3D^42^, and CDD^43^, were analyzed through InterProScan. The functional annotation results showed that 25,309 genes (96.13%) matched UniProtKB records, with 5,114 genes labeled as “uncharacterized protein” and 1,040 genes labeled as “unknown function”. Through the integration of InterProScan and eggNOG annotations, HapA contained 16,889 genes assigned to COG functional categories, 14,974 genes with GO annotations, 7,291 genes with KEGG pathway annotations, and 4,719 genes with Enzyme Codes (EC numbers) (Table 5).

## Data Records

The raw sequencing data and genome assembly of *Folsomia candida* have been deposited at the National Center for Biotechnology Information (NCBI). The PacBio HiFi, ONT ultralong, and ONT long-read transcriptome can be found under identification numbers SRR35197880^44^, SRR35197879^45^, and SRR35197878^46^, respectively, under the BioProject accession number PRJNA1040973 and BioSample accession number SAMN17109001. The Hi-C data of *Folsomia candida* DK can be found under the identification number SRR13435515^47^ under the BioProject accession number PRJNA686204. The Illumina transcriptome short reads can be found under the identification number SRR13452035^48^ under the BioProject PRJNA686207. The assembled haplotype-resolved genomes have been deposited in the GenBank in NCBI under accession numbers GCA_052721305.1^49^ (HapA) and GCA_052721315.1^50^ (HapB). The annotation results for repeated sequences, gene structure, and functional prediction have been deposited in the Figshare^51^ database.

## Technical Validation

Two methods were used to evaluate the quality of the genome assembly. Firstly, BUSCO v5.4.4^52^ was applied for assembly completeness calculation with the reference Arthropoda gene set (n = 1,013) with the euk_genome_met mode. The final genome assembly showed a BUSCO completeness of 97.5% for both haplotypes, including 972 (96.0%) single-copy BUSCOs, 15 (1.5%) duplicated BUSCOs, 7 (0.7%) fragmented BUSCOs, and 19 (1.8%) missing BUSCOs. The final annotation validation of HapA was also calculated by BUSCOs with a protein mode with the reference Arthropoda gene set (n = 1,013). The final annotated HapA also exhibited a BUSCO completeness of 98.1%, including 833 (82.2%) single-copy BUSCOs, 161 (15.9%) duplicated BUSCOs, 7 (0.7%) fragmented BUSCOs, and 12 (1.2%) missing BUSCOs. To investigate the quality of the *de novo* assembly, Merqury v1.3^53^ was performed to identify possible assembly sequence errors based on efficient k-mer set operations and QV score calculation. Consequently, the k-mer completeness value of the HapA is 99.03%, and the QV score is 55.57. For HapB, the k-mer completeness value is 98.97% and the QV score is 54.67. Both the k-mer value and the QV score reflect the high accuracy of the base pairs, combined with the BUSCOs, which exhibit the high completeness and accuracy of our genome assembly.

## Data Availability

All data used in this manuscript can be found under the following list: PacBio HiFi reads: SRR35197880^44^. ONT-Ultralong reads: SRR35197879^45^ ONT-long transcript reads: SRR35197878^46^ Hi-C data: SRR13435515^47^ Illumina short transcript reads: SRR13452035^48^ HapA genome assembly: GCA_052721305.1^49^ HapB genome assembly: GCA_052721315.1^50^. Genome annotation information: 10.6084/m9.figshare.30173248.v1.

## Code availability

No special scripts or code were developed during this work. All computational analyses were conducted using established bioinformatics software with versions and key parameters specified in the Methods section. Default parameters were used unless otherwise indicated.
